# Eliciting tacit knowledge: The potential of a reflective approach to video-stimulated interviewing

**DOI:** 10.1007/s40037-018-0487-9

**Published:** 2018-11-16

**Authors:** Marije van Braak, Esther de Groot, Mario Veen, Lisanne Welink, Esther Giroldi

**Affiliations:** 1 Erasmus Medical Center, Rotterdam, The Netherlands; 2 University Medical Center Utrecht, Utrecht, The Netherlands; 3 Maastricht University, Maastricht, The Netherlands

**Keywords:** Video-stimulated interviewing, Recall, Reflection, Medical education research

A Qualitative Space highlights research approaches that push readers and scholars deeper into qualitative methods and methodologies. Contributors to A Qualitative Space may: advance new ideas about qualitative methodologies, methods, and/or techniques; debate current and historical trends in qualitative research; craft and share nuanced reflections on how data collection methods should be revised or modified; reflect on the epistemological bases of qualitative research; or argue that some qualitative practices should end. Share your thoughts on Twitter using the hashtag: #aqualspace

## Introduction

‘How to know what others know?’ is a pertinent question debated extensively in education [1]. The *tacit knowledge of professionals*, the seldom expressed knowledge that guides professional practice [2, 3], has received particularly ample attention [4] for good reason. Explicating tacit knowledge can aid professionalization in several ways: it can improve performance by encouraging professionals to reflect on their behaviour; it can help communicate knowledge to others; it can facilitate evaluation by linking aspects of behaviour to outcomes of that behaviour; and finally it can aid construction of ‘artefacts’ that can assist in daily practice [5]. Each of these benefits underscores the exigency of explicating the tacit knowledge of professionals, e. g., practitioners (whether or not in training) and teachers in the field of medical education (cf. [6]).

But how to elicit the tacit knowledge that informs professional practice? Mainstream research tools such as surveys and formal interviews, though employed extensively, are limited with respect to eliciting tacit knowledge. Formal interview questions such as ‘What do you aim for in your teaching?’ might be too abstract to answer and difficult to address given that they concern understandings that may rarely get expressed [7]. It has been argued that we need elicitation techniques that prompt participants to express their tacit knowledge by displaying the focus of interviews onto external stimuli, either visual, verbal, or written [7, 8]. These stimuli can facilitate the articulation of complex ideas in the participant’s own conceptual categories, bringing to the fore understandings that would otherwise remain below the surface [7]. Despite their clear value, however, many elicitation techniques use stimuli that are only distantly related to actual practice (e. g. photo elicitation, drawings) or interfere with the very practices that are under investigation (e. g. think-aloud protocols) [7, 9]. Video-stimulated interviewing [10] (VSI, also known as video elicitation interviewing [11]) is a promising exception that avoids both disadvantages by having participants view a video of their own behaviour while being asked questions about the recorded situation [12]. As such, VSI is a promising tool for eliciting the tacit knowledge that informs professional practice, provided that, as we argue in this paper, the tool is used to stimulate *reflection*, not *recall*.

Historically, VSI has been used to elicit accounts of participants’ thinking at the time of recording (hence the frequent mentions of the tool as video-stimulated *recall* interviewing) [7]. Notwithstanding its potential value to other research purposes, this approach to VSI is problematic in research aimed at eliciting tacit knowledge. Indeed, we argue, a *reflective *approach to VSI is more productive to that aim. In the following, we first place both approaches in historical context to show their distinct epistemological roots. We then explain why recall VSI is less suited to uncovering tacit knowledge and why reflective VSI is better suited to do so, drawing on research outside of medical education, the few examples of reflective VSI in medical education, and from our own experience. Finally, we discuss several challenges and best practices of reflective VSI. This will help researchers to unleash the potential of reflective VSI in their own medical educational research, facilitating the search for answers to pressing issues that will aid progress in research and benefit implementation.

## The historical context of recall and reflective VSI

Set in educational research, video-stimulated interviewing was introduced as a qualitative technique that facilitates a subject’s reliving of a situation ‘with vividness and accuracy if […] presented with a large number of the cues or stimuli which occurred during the original situation’ [13, p. 161]. Early applications of VSI appeared in the 1960s [14]. With the rise of cognitive psychology, many researchers used VSI to elicit reports of thinking (see Tab. 1; [9, 15]). Most of this research was conducted in general education settings (for an overview see [16]) and physician-patient interactions (see [11]). The research was based on the assumption that people have access to their internal thought processes at some level, that people can verbalize these (i. e., that *reliving* is a valid approach to stimulate accurate *recall*), and that these verbalizations are retrievable through video stimulation. This assumption, however, came under fire with the rise of constructivist thinking.

**Table 1:** A comparative summary of recall VSI and reflective VSI

	Recall VSI	Reflective VSI
*Historical context*	Post-positivism, cognitive psychology [9, 15]	Interpretivism, constructivist thinking [9, 16]
*Aim*	Gain insight into cognitive processes underlying and taking place during actual behaviour [7]	Produce an interpretation of a phenomenon (behaviour, practice) as the participant understands it [9]
*Procedure*	Stimulate participants’ retrospective description of their cognitive processes [7]	Stimulate the participants’ retrospective reflections on their situational understanding, routine procedures, and intuitive decision-making [4, 42]
*Sample research questions*	‘What factors influence physicians’ decisions to discuss smoking cessation with patients?’ [40] ‘What processes and stages of treatment decision-making do women with early stage breast cancer perceive?’ [41]	‘Why do physicians communicate with their patients about medication use and adherence the way they do?’ [30] ‘How does student nurses’ reflective learning develop in the context of health counselling and promotion in the clinical training section of a 3-year nursing education program?’ [26]
*Sample interview questions*	‘What do you think of [behaviour, event]?’ [12] ‘What were you thinking when you decided to [behaviour]?’ [12]	‘How would you evaluate [behaviour, event]?’ ‘What do you notice when you watch [behaviour, event]?’

In the interpretivist research paradigm to which constructivism belongs, the aim of research applying VSI is not to introspectively elicit in-the-moment processes of thinking, problem-solving or decision-making. Instead, the researcher’s intention is ‘to produce an interpretation of the phenomenon as the informants conceive and understand it’ [9, p. 185] (see Tab. 1). The focus is not on *recall* and the need for maximum validity and reduced researcher interference, as it is in post-positivist approaches to VSI. Instead, in constructivist approaches the focus is on *reflection *through constructing meaning and the need to respect the essential role of the researcher in producing the interpretation of the reality under investigation [9, 17]. From this perspective, the technique is suited to stimulate participants to give meaning to their behaviour, allowing for the unfolding of implicit theories, expectations, strategies and views [18]. Research using reflective VSI has only recently begun to emerge [16] and is scattered in educational research in general, but especially in medical education. It is in the interpretivist approach to VSI that we find the underexplored potential of VSI for eliciting tacit knowledge in medical education research. Before arguing for this potential of reflective VSI, we first discuss why recall VSI is less suited to eliciting tacit knowledge.

## Why recall VSI is limited in eliciting tacit knowledge

If one’s aim is to elicit tacit knowledge in the context of medical education, the recall approach to VSI is problematic for two reasons. First, recalled *cognitive processes* are not necessarily linked to the (often general) tacit knowledge that drove the actual behaviour. What one thinks *during *teaching, for example, does not necessarily relate to what one thinks *about *teaching. What one thinks *during* a patient consultation does not necessarily reflect one’s ideas *about* patient consultations. Even if retrospective reports of thinking could reveal hints of tacit knowledge, stimulating concurrent cognitive processes to elicit generalized unspoken ideas is doubtful at best and invalid at worst.

The second issue with recall VSI is more general but fundamental to our evaluation of recall VSI as a tool to elicit tacit knowledge. The extent to which recalled thoughts accurately reflect thoughts that occur during the recorded event is unclear and widely criticized [7, 15]. Participants might consciously censor the recall while being interviewed [19] or unintentionally involve sense-making processes that produce convincing stories unrelated to their thinking processes during the event [9]. Despite ‘mov[ing] analysis from a generalized response (…) to a specific, empirical situated focus, where the observed reality challenges the tendency to provide moral or ideal accounts’ [20, p. 9], recall VSI cannot warrant capturing participants’ past thinking [7, 21]. In fact, the ‘luxury of meta-analysis and reflection’ [15, p. 271], which is absent at the time of recording, cannot but evoke interference in present thinking in reported verbalizations [7]. Various measures have been proposed to optimize recall accuracy, such as minimizing the time delay between event and recall and using the right type of (non-directive) question probes [22, 23]. Even then, though, we ought to be aware that applications of recall VSI merely yield retrospectively recalled, potentially unreliable memories that are possibly coloured by post-event reflections on particular, but not necessarily conscious behaviour.

If recall is unlikely to be accurate and thinking is only remotely linked to tacit knowledge, then recall VSI is not very well suited to eliciting tacit knowledge. Reflective VSI departs from another viewpoint, asks other questions, and provides different output, making it, as we argue in the next section, better suited to uncovering the tacit knowledge that informs professional practice in medical education.

## How reflective VSI can elicit tacit knowledge

In contrast to recall VSI, reflective VSI asks participants to *make sense* of their own behaviour [7, 21, 24, 25]. This sense-making process, though grounded in a specific context, transcends the specific behaviour that occurred during the recording. The specific behaviour merely triggers participants to give meaning to their behaviour in general. By ‘prompting explanation and justification of practices’ reflective VSI spurs reflection on practice [7, p. 196]. Importantly, participants’ reflections thus elicited are interpreted as constructed *in the moment of interviewing* [24]. Whether or not the reflections resemble cognitive processes that occurred in the recorded situation is not an issue in this interpretivist paradigm [9] and does not change the researcher’s interpretation of the reflections.

Medical education research applying VSI as a reflection-stimulating tool is sparse. Those studies that explicitly report eliciting reflection with videos feature two uses of reflective VSI. First, some studies use reflective VSI as a way to investigate *reflection on (professional) behaviour* [7]. Liimatainen et al., for example, analyzed reflectivity levels in student nurses’ reflections on their videotaped counselling situations at different time points to gain insight into reflective skills of nursing students over time [26]. They describe their interviews with the student nurses as reflection-on-action situations. Such situations, they argue, bring to the fore the ‘students’ personal ways of seeing phenomena and their interrelationships’ [26, p. 651]. Similarly, Hewson had an attending physician and a resident in a regular staffing episode reflect on their videorecorded interaction in the context of professional training of physicians [27]. Participants of the study were invited ‘to stop [the recording] at any time to reflect on [their] thoughts about what was happening’ [27, p. 228]. This yielded both retrospectively reported thoughts and reflections-on-action, which appeared beneficial to the professional training of the medical staff involved.

In both studies, eliciting tacit knowledge was not mentioned as the primary aim. We now show two of our own studies as examples of how medical education research using reflective VSI can uncover tacit knowledge. In one of our current research projects we use video-recordings of educational sessions for general practitioners in training to stimulate teachers of the recorded sessions to reflect on the tacit knowledge that guides their behaviour when they teach these sessions. These interviewee contributions can be seen as descriptions of professional craft knowledge [18]. When shared with teachers, these descriptions can facilitate teacher professionalization [5]. In another study, we asked clinician pharmacists to reflect on boundary-crossing conversations with their supervising general practitioner [28]. In both studies, we were interested in participants’ *actual reflection on particular professional behaviour. *The reflective VSI functioned accordingly, stimulating reflective discussion on the issues of interest.

Besides the use of reflective VSI to investigate reflection on professional behaviour, the medical education literature features a study that used reflective VSI to shed light on the largely tacit* mechanisms behind behaviour* [7]. These mechanisms frequently remain implicit in recall of behaviour, but can be verbalized as participants make sense of recorded episodes of that behaviour [7, 29]. Van Dulmen and Van Bijnen used reflective VSI to investigate why general practitioners might refrain from talking to patients about medication use and adherence [30]. Analyzing general practitioners’ reflections on a recorded visit that included segments about medication use and adherence, they identified various determinants that influence general practitioners’ communication behaviour, thus revealing the general practitioners’ tacit knowledge about a particular aspect of consultation communication.

Likewise, in an ongoing research project of our own, we use consultation recordings to ask general practitioners (in training) to reflect on the role of evidence in their decision-making. Since evidence-based decision-making is often regarded as a normative issue, using the recordings might stop the professionals from giving socially acceptable answers instead of reflecting on their actual behaviour. In another project, we used videotaped consultations in interviews with experienced doctors to stimulate them to reflect on their reassuring behaviour [31]. The level of detail in the doctors’ reflections allowed us to uncover working mechanisms underlying the reassuring behaviour, guiding the reader to put this behaviour in context.

In each of the reflective VSI examples just described, researchers avoid the pitfall of assuming that interviewees’ utterances provide a window to their former thought processes. Rather, they view the reflective interview as an opportunity (for interviewees) to ‘review events in which they have participated from an outsiders’ perspective but with an insider’s insight into their motivations and intentions’ [32]. The focus on reflection enables participants to construct meaning to behaviours applied unconsciously in practice [31], thus making the tool especially suited to uncover tacit theories, implicit ideas, and unspoken strategies behind learning and teaching in medical education.

## Critical comments on reflective VSI

Although reflective VSI, as we have just argued, is well suited to elicit tacit knowledge in medical education, several characteristics make the tool either more or less apt for application to aims that particular studies might have. On a positive note, reflective VSI offers rich learning opportunities for participants [7, 25]. In our own studies as well as in at least one study mentioned above [27] participants expressed the value of receiving direct feedback on their professional competencies (such as clinical communication skills and teaching practices) outside the assessment context. Data resulting from applying reflective VSI can inform continuous professional development, provided that data collection follows appropriate informed consent procedures. In a time when researchers struggle to recruit enough participants, these learning opportunities serve as an incentive to participation. Thus, if the aim is to elicit tacit knowledge to facilitate learning, develop training courses, or improve education, the reflective VSI tool is particularly suitable.

However, reflective VSI also has its challenges. One possible risk is that participants can feel vulnerable and/or ‘judged’ [21]. In our work with triage nurses, for example, nurses were accustomed to listening to the recordings in an assessment setting with their supervisor. During the interviews, we had to assure participants repeatedly that they were not being judged, but that we were interested in their decision-making in a very complex context. In dealing with this issue, it is helpful to consider that (a) participants are more open and more likely to reflect on their emotions when interviewed by a peer, especially if that peer is less experienced [20, 33]; (b) the interviewer should not be involved in the participants’ medical/professional training; and (c) it is important to repeatedly emphasize the goal of the study and that the participant will not be assessed [31, 34].

Second, time issues might arise if short extracts of recorded material facilitate extensive reflection [11]. The researcher might pre-select fragments of interest to show during the interview. Alternatively, during the interview the researcher could invite interviewees to select the fragments to keep data collection as participant-centred as possible. As Barton notes, ‘ [G]iving participants greater control can also yield data that more authentically reflect their conceptual categories. […] Although researchers may believe that they can assemble tasks that allow perspectives to emerge, asking participants themselves to contribute to the process makes this more likely.’ [7, p. 182–3]. If the recorded material is short, one might consider playing the material from start to end. In any case, carefully considering data selection and presentation is vital to aligning data collection design with the aim of the study.

At the other end of the spectrum, participants might struggle to produce reflections [21]. In our ongoing research with general practice teachers, for example, teachers sometimes find it hard to let go of describing their then-occurring thoughts and focus on constructing in-the-moment reflections (but see [35] for a report on participants’ tendency to reflect instead of recall). The researcher needs to be prepared to cope with this situation. Preparing reflection-stimulating, non-directive prompts and offering these if necessary will guard the success of reflective VSI.

Given these considerations, reflective VSI might not always be the best tool to uncover tacit knowledge. It might not be suitable for participant groups that, for whatever reason, have difficulty with reflecting. These participant groups require methods that are less demanding such that data collection is not confounded by the method itself. And secondly, it might not be suitable for research projects with limited time frames or budgets. Such projects require less challenging methods in terms of data collection time, resources, and ethical approval (for a discussion on ethical approval, see [36]).

For other medical educational research aiming to elicit tacit knowledge, however, we encourage researchers to explore the potential of reflective VSI. Reflective VSI is a promising option for research that seeks to produce participants’ interpretations of a situation, since it allows participants to construct meaning and interpret reality as the recording is played. Reflective VSI is a promising option for research with professionals as participants, since it is well suited to uncover implicit theories, unconsciously made choices, expectations, expert strategies, and individual views [3]. And finally, reflective VSI is a promising option for research that aims to serve applied educational purposes [7, 21], since it can help participants develop useful insights into their own participation, interaction, and behaviour in the settings involved.

## Best practice in reflective VSI

To aid medical education researchers to unleash the potential of reflective VSI in their research, a few comments on best practice are in place. Discussions on applying reflective VSI (although not always named as such) outside the medical education field offer various suggestions for good practice [9, 11, 21, 24, 30, 37–39]. From these discussions, we have identified three best practices.

First, the researcher should *acknowledge their own non-neutrality*, since researcher background and perceptions (in fact, their own tacit knowledge) influence meaning construction during the interview [9]. As the aim is to produce the participant’s interpretation of the recorded situation, the researcher’s task is to assist the interviewee as an expert participant in the situation. As such, the researcher should listen actively, ask for clarification if needed, and avoid leading and evaluative questions [9]. Acknowledging one’s own non-neutrality by actively considering steps to minimize biased interview guidance is core to this interview attitude. To envisage how that might work in practice, consider Rowe’s reflection on her role as a music education researcher: ‘The researcher in this study, who is also a piano teacher, was conscious of the necessity to withhold her musical and pedagogical reactions when observing the lessons and to focus on the interactions between teacher and pupil. Similarly, when interviewing the pupils she had to avoid falling into her accustomed ‘teacher’ role with its accompanying assumptions of power over a child’ [21, p. 428]. By being aware of her own bias and formulating behaviour to minimize its influence on the interview, this researcher acknowledged and dealt with her own non-neutrality.

Second, the researcher should *use apt prompts* [11, 24]. Apt prompting minimizes disturbance of sense-making processes during the interview [37]. Apt prompts in reflective VSI ‘remain “neutral” while providing a context e. g. study aim or orientation for the participant to comment’ [17, p. 16]. A prompt, then, should only hint at a particular interpretation of a situation if the participant gives reason to assume that interpretation. Besides, prompts should be free of formulations that induce interviewees to try to recall their cognitive processes during the recorded event (e. g., ‘What did you think when X happened?’). As an example, Tab. 2 presents the prompt types we used for our interviews with teachers in general practice training in order of minimal to most directed guidance. We used the more guiding prompt types only when less guiding prompts did not yield the desired reflection, as the more guiding prompts risk potentially disturbing the participant’s sense-making process.

**Table 2:** Types of prompts for interviewers

Stop the recording and …
1. Remain silent;
2. Give a neutral description of something in the recording (e. g., ‘You are saying X here.’);
3. Ask a neutral, open question (e. g., ‘What is happening here?’);
4. Present an observation (e. g., ‘You appear caught off-guard at this point.’);
5. Ask for intentions/aims (e. g., ‘What did you achieve with X?’);
6. Ask an evaluative question (e. g., ‘What do you think of X?’).

Apt prompting, however essential it may be, appears to be difficult to implement. It is an unnatural form of communication, since we do not usually remain silent or unresponsive if our aim is to elicit talk in ordinary conversation. Our everyday inclination is to *ask *for an *answer* and not burden the responder with finding out what we are asking for. Apt prompting, thus, requires systematic practice on the part of the researcher. Nevertheless, we encourage researchers to carefully prepare a similar set of non-leading prompts and include them in an interview protocol. They structure the interview and will likely produce rich data for the study’s research question [11, 17, 37].

Third, in the analysis phase the researcher should *view the interview as an opportunity for collaborative meaning construction* [24, 39]. As Holstein and Gubrium state, ‘[I]nteractional, interpretative activity is a hallmark of all interviews’ [38]. Especially when the interview is meant to uncover participants’ sense-making of particular situations, analyzing the interview as a collaborative achievement [39] between researcher and participant is crucial. Merely analyzing participant responses ignores the researcher’s part in the collaborative construction. Consider, for example, the extract of an interview with one of the teachers (T) of our research into a particular teaching practice in General Practice training (Tab. 3).

**Table 3:** Interview extract

1	I	And did that influence your behaviour in this case?
2	T	Eh no, I thought: just let them talk for a moment
3	I	You just let them talk for a moment
4	T	[…] And sometimes it’s good to let residents tell the
5		story in detail, because I also think that, you know,
6		that also makes the experience- experiences come
7		more to life so that we can discuss it with each other.
8		So that it isn’t just a story, with some dry facts. That’s
9		why I think it’s also important to give feedback
10	I	Ok. So that others can imagine it too
11	T	Yes
12	I	Then it gets more lively?
13	T	It gets livelier, yes
14	I	And that makes giving feedback easier?
15	T	It fits better
16	I	It fits better, ok
17	T	Yes
18	I	Fits what actually happened, you mean?
19	T	Yes

In this extract, the teacher and interviewer construct meaning collaboratively. The interviewer summarizes the teacher’s contribution and produces two interpretations to check her understanding of the teacher’s interpretation of the situation (lines 10, 12, 14). The interpretation in line 14 elicits a slightly different interpretation from the teacher, which is clarified before this meaning-construction sequence is closed. Analyzing (and presenting) the contributions of the teacher without displaying the interviewer’s contributions or summarizing the teacher’s turns in a cogent quote would yield an incomplete representation of the interaction. What is more, such representation would not do justice to the source of certain interpretations: it would blur the interviewer’s and interviewee’s understandings of the situation discussed. When the researcher’s contributions and possible influences are not made explicit while analyzing, the pitfall of attributing the interviewer’s interpretations to the interviewee looms large, hampering an accurate analysis of the interviewee’s meaning-construction process.

## Conclusion

Reflective VSI, unlike traditional applications of VSI as a tool to stimulate recall, can benefit medical education researchers who aim to uncover participants’ implicit theories, reflections on key events, or seemingly mundane, possibly unconscious, routines in a real situation of interest. By eliciting this tacit knowledge, reflective VSI captures the knowledge and expertise of educators, medical professionals, medical students, residents, and patients for later access by, for example, inexperienced professionals. Reflective VSI can also serve a powerful practical purpose, as it can help participants develop insights into their own participation, interaction, and behaviour in the settings involved, ultimately leading to improved medical or educational conduct.

Researchers applying reflective VSI, however, will encounter several hurdles along the way. For a good application of the reflective VSI tool, researchers should make explicit their own non-neutrality, design apt interview prompts, and analyze the interview as collaborative meaning construction. Each of these measures strengthens reflection on past experience. These reflections can help us find answers to questions about the tacit knowledge possessed by others, thus making reflective VSI a powerful tool in the continuing debate on ‘how to know what others know’.
